# Visually Appealing iEat® Food Products Enhance Visual Cortex Activity in Patients with Dysphagia: A Functional MRI (fMRI) Study

**DOI:** 10.7759/cureus.92249

**Published:** 2025-09-13

**Authors:** Keishi Okamoto, Satoshi Tanaka, Yasutaka Suzuki, Syuya Kato, Takashi Shigematsu, Kenjiro Kunieda, Ichiro Fujishima

**Affiliations:** 1 Department of Rehabilitation, Hamamatsu City Rehabilitation Hospital, Shizuoka, JPN; 2 Department of Psychology, Hamamatsu University School of Medicine, Shizuoka, JPN; 3 Department of Radiological Technology, Seirei Mikatahara General Hospital, Shizuoka, JPN; 4 Department of Radiological Technology, Hamamatsu City Rehabilitation Hospital, Shizuoka, JPN; 5 Department of Rehabilitation Medicine, Hamamatsu City Rehabilitation Hospital, Shizuoka, JPN; 6 Department of Neurology, Gifu University Graduate School of Medicine, Gifu, JPN

**Keywords:** appetite, dysphagia, food appearance, modified diets, mri

## Abstract

Objective: Texture-modified diets are common for patients with dysphagia, but can reduce food recognition and appetite. iEat® foods maintain the appearance and nutritional value of regular foods while being softened. This study aimed to investigate the brain activity in patients with dysphagia while viewing visually appealing iEat® food products (EN Otsuka Pharmaceutical Co. Ltd, Tokyo, Japan) using functional magnetic resonance imaging (fMRI).

Methods: This cross-sectional neuroimaging study was conducted at a rehabilitation hospital from January 2017 to December 2020. Twenty patients with dysphagia were recruited through convenience sampling from inpatients admitted to the hospital. Patients with dysphagia were presented with images of iEat food products, pureed foods, and non-food objects while their brain activity was measured using fMRI.

Results: The presentation of iEat food products elicited significantly higher subjective appetite ratings compared to pureed foods. Viewing iEat food products significantly increased activity in the visual cortex, particularly in the left middle occipital gyrus. Additionally, correlation analysis revealed a significant positive relationship between the left middle occipital gyrus activity and subjective appetite ratings. No significant activity was observed in the reward system, including the amygdala, orbitofrontal cortex, and striatum.

Conclusions: The findings suggest that visually appealing food presentation, such as iEat food products, enhances visual cortex activity in patients with dysphagia, potentially contributing to improved appetite and food intake.

## Introduction

Various sensory stimuli, such as vision, taste, and smell, trigger anticipatory physiological responses that contribute to the proper metabolism of food. For patients with dysphagia, modifying food texture is a widely used approach to facilitate oral intake [1]. However, this process can obscure the food's original appearance, which may negatively impact patients' appetite and quality of life (QOL) [2,3]. Research has associated texture-modified diets with decreased appetite in older adult patients with dysphagia [4]. Conversely, studies have demonstrated that appealing food presentation can improve satisfaction in healthy older adults [5] and boost food intake in hospitalized individuals [6].

iEat® (EN Otsuka Pharmaceutical Co. Ltd, Tokyo, Japan), an innovative food product, retains the appealing appearance, taste, and nutritional value of regular foods while being softened using an enzymatic homogenous osmosis process, which allows for easy oral breakdown [7-9]. This texture-modified food provides a new dietary option for individuals with swallowing impairments [10,11]. Questionnaire-based studies have demonstrated that patients report greater satisfaction with the appearance of iEat food products compared to pureed options [7,12], suggesting that the visually appealing nature of iEat products may enhance patients' appetite and food intake behavior. In a previous functional magnetic resonance imaging (fMRI) study, we investigated the effect of visually different meals on brain activity in healthy adults [13]. iEat products elicited significantly higher subjective appetite ratings compared to pureed foods, and brain activity revealed significant activation not only in the reward system, including the amygdala, striatum, and orbitofrontal cortex (OFC), but also in the visual cortex [13]. The activation of both reward and visual processing regions suggests that the appealing appearance of iEat food products may engage multiple neural mechanisms to enhance appetite and food-related responses.

We hypothesized that the visually appealing nature of iEat food products would engage the reward system and visual cortex in patients with dysphagia, extending our previous findings in healthy adults. Using fMRI, this study tests this hypothesis, aiming to deepen our understanding of the neural processes involved in perceiving appealing foods in individuals with swallowing difficulties.

## Materials and methods

This was a cross-sectional neuroimaging study conducted at Hamamatsu City Rehabilitation Hospital, Japan, from January 2017 to December 2020. The study was approved by the Ethics Committee of Hamamatsu City Rehabilitation Hospital (approval number: 16-79) and adhered to the Declaration of Helsinki's ethical standards. All participants provided written informed consent before participating.

Participants

Twenty patients with dysphagia were recruited through convenience sampling from inpatients admitted to the hospital. Participants were recruited from patients diagnosed with dysphagia by rehabilitation physicians and prescribed dysphagia rehabilitation by speech-language hearing therapists. Participants were selected based on their ability to consume a texture-modified diet, which aligns with the texture of iEat food products. This selection criterion ensured that all participants could safely consume the study foods. The sample size was determined based on the previous fMRI study in healthy individuals [13]. Inclusion criteria were: (1) eating pureed or minced wet food, (2) no contraindications for MRI, (3) no severe communication impairments interfering with study participation, (4) absence of observable lesions in the frontal lobe, striatum, amygdala, insular cortex, or gustatory cortex (confirmed by CT or MRI) to ensure effective task engagement and reliable performance.

Stimuli

**Figure 1:**
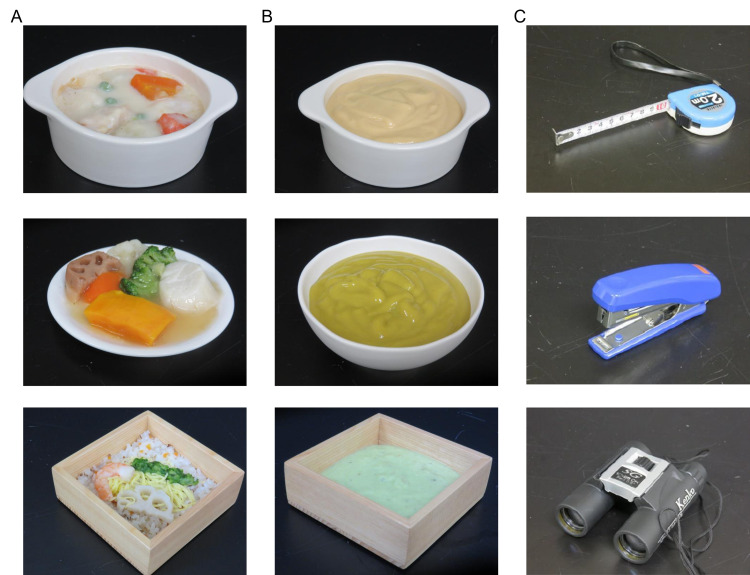
Visual stimuli used in the fMRI experiment Visual stimuli examples utilized in the fMRI study. (A) iEat® food images (EN Otsuka Pharmaceutical Co. Ltd, Tokyo, Japan) showing gratin, scallops and vegetables in starch sauce, and scattered sushi from top to bottom, respectively. (B) Pureed food images displaying blended versions of each corresponding iEat food item from panel (A). (C) Non-food object images featuring measuring tape, stapler, and binoculars from top to bottom, respectively. Image Credit: Authors

For the fMRI study, participants observed visual stimuli from three categories: iEat food products, pureed foods, and non-food objects. The iEat food stimuli comprised 30 images displaying diverse dishes, such as gratin, scallops, and vegetables in starch sauce, and scattered sushi (Figure 1A). Pureed food stimuli were generated by blending the iEat food products and presenting 30 pictures of the resulting purees (Figure 1B). Non-food object stimuli consisted of 30 photographs featuring common household items, including measuring tape, stapler, and binoculars (Figure 1C). All stimuli were displayed in color with a resolution of 4608 × 3456 pixels. The images were presented on an LCD monitor (LCD32, NordicNeuroLab AS, Bergen, Norway) using Presentation software (Neurobehavioral Systems Inc., Berkeley, California, United States) and viewed by participants via a mirror, ensuring a consistent visual angle of 1°.

Study procedure and tools used

Before the experiment, the patients' feeding status was assessed using the Food Intake LEVEL Scale (FILS) [14], and their cognitive function was evaluated using the Mini-Mental State Examination-Japanese (MMSE-J) [15]. The MMSE-J instruments had been purchased by our hospital. Participants fasted for at least five hours before the fMRI to ensure optimal neural responses to food stimuli [16]. During fMRI, participants viewed images from each category and mentally assessed their desire to consume the foods, without any motor response. Each image was shown for 3.0 seconds, followed by a 1.0-second blank interval. The fMRI sessions comprised alternating 30-second rest and picture-viewing conditions, with each visual condition presented three times per session. The experimental design consisted of alternating blocks: a 30-second visual presentation condition followed by a 30-second rest period. The order of visual presentation for each category (iEat food products, pureed foods, non-food objects) was counterbalanced among participants. Following the fMRI sessions, high-resolution anatomical MRI data were acquired. Subsequently, participants completed subjective appetite ratings outside the scanner, evaluating how much the iEat and pureed food images had stimulated their desire to eat using a 10-point Likert scale (1 = no appetite stimulation, 10 = maximum appetite stimulation). Appetite was evaluated using the Japanese version of the Council on Nutrition Appetite Questionnaire (CNAQ-J) [17,18]. Permission has also been taken from the authors of the original CNAQ/Simplified Nutritional Appetite Questionnaire (SNAQ) via email.

Data collection

fMRI data were obtained using a 1.5 Tesla MRI scanner (Optima MR360-Advance; GE Healthcare Technologies, Inc., Chicago, Illinois, United States) with echo-planar imaging (EPI) sequences. The EPI parameters were as follows: repetition time (TR) = 2500 ms, echo time (TE) = 40 ms, flip angle (FA) = 85°, field of view (FOV) = 192 mm, voxel size = 3.0 × 3.0 × 4.0 mm, and 33 axial slices. Anatomical data were acquired using a 3D fast spoiled gradient-echo sequence.

Image processing and analysis were performed using SPM12 software (Wellcome Department of Cognitive Neurology, University College London, London, United Kingdom). The functional data were realigned to the mean functional image to correct for head motion, normalized to the Montreal Neurological Institute (MNI) template [19], and spatially smoothed using a Gaussian kernel with a full-width half-maximum (FWHM) of 8 mm.

Data analysis

Statistical analyses were conducted using a two-level approach. At the first level, individual activation patterns for each visual category were evaluated with a general linear model [20]. Picture stimuli were modeled as regressors using boxcar functions convolved with the canonical hemodynamic response function. Motion parameters were incorporated as covariates of no interest. Areas showing higher activation during picture presentations compared to rest periods were identified. iEat food-specific brain activity was determined by subtracting images acquired during pureed food and non-food object presentations from those acquired during iEat food presentations. Second, individual contrast images were entered into a random effects group analysis [21]. For each contrast, one-sample t-tests were conducted, with a statistical threshold of family-wise error (FWE)-corrected p < 0.05 at the voxel and cluster levels for the whole brain or small volumes around regions of interest (ROI) coordinates.

For the ROI analysis, spherical ROIs (radius = 10 mm) were created in the left OFC (x = −28, y = 30, z = −18), left amygdala (x = −24, y = 0, z = −22), left ventral striatum (x = −12, y = 8, z = 0), right middle occipital gyrus (x = 32, y = -80, z = 14), right fusiform gyrus (x = 42, y = -60, z = −16) and left lingual gyrus (x = −18, y = -84, z = −14) based on a previous study in healthy adults [13]. Following reviewer recommendations, we conducted additional ROI analyses to more comprehensively evaluate reward-related brain activity in the following regions: right OFC (x = 28, y = 30, z = −18), right amygdala (x = 24, y = 0, z = −22), right ventral striatum (x = 12, y = 8, z = 0), left anterior insula (x = -38, y = 5, z = −8) and right anterior insula (x = 40, y = 6, z = −10) based on previous studies [13,22].

## Results

Patient demographics

Twenty patients with dysphagia, with a mean age of 70.7 years (SD ± 12.5) and including two female patients, participated in this study. Table 1 details patient demographics. Mean education level was 13.1 ± 2.6 years, BMI was 19.7 ± 3.9 kg/m^2^, and MMSE-J score was 26.9 ± 2.7. The median FILS score was 7 (interquartile range (IQR): 7-8). Underlying conditions varied; nine patients had cerebrovascular disease (five cerebral infarction, four cerebral hemorrhage), six patients had dysphagia with aspiration pneumonia, and individual cases included spinal muscular atrophy, post-radiation nasopharyngeal cancer, glossopharyngeal and vagus nerve paralysis, mixed laryngeal paralysis, and disuse syndrome.

**Table 1:** Demographics and clinical characteristics of the patients included in the study (N=20) M: Male; F: Female; R: Right; L: Left; MMSE-J: Mini-Mental State Examination-Japanese; FILS: Food Intake LEVEL Scale *Data given as mean±SD for the total scores MMSE-J instruments were purchased by our hospital and used with permission.

ID	Sex	Age (years)	Education (years)	Diagnosis	Onset (days)	BMI	MMSE-J	FILS
1	M	72	9	Mixed laryngeal paralysis	220	15.3	24	8
2	M	79	16	Dysphagia with Aspiration pneumonia	71	21	28	8
3	M	70	18	Dysphagia with Aspiration pneumonia	63	21.2	27	5
4	M	68	12	R lateral medullary infarction	508	18.7	23	5
5	M	62	16	Spinal muscular atrophy	153	17	29	5
6	M	76	9	L pons branch atheromatous disease	61	22.7	27	7
7	M	55	12	R putaminal hemorrhage	26	22.2	28	8
8	M	70	16	Dysphagia with Aspiration pneumonia	32	17.7	30	8
9	M	48	12	R frontal subcortical hemorrhage	54	30.8	22	8
10	M	86	12	Dysphagia with Aspiration pneumonia	81	18.1	28	7
11	F	42	14	Nasopharyngeal cancer post-radiation	1121	16.6	30	8
12	M	70	12	R lateral medullary infarction	326	22.1	24	7
13	M	88	16	Dysphagia with Aspiration pneumonia	101	16	28	7
14	M	82	9	Dysphagia with Aspiration pneumonia	114	14.4	27	8
15	M	67	12	L lateral medullary infarction	1147	24	30	8
16	M	77	12	Glossopharyngeal paralysis	147	17.4	28	7
17	M	74	16	R vertebral artery dissection	124	21.5	30	8
18	F	60	14	L medullary hem.	494	15.3	29	8
19	M	87	12	L lateral medullary infarction	42	23.5	23	7
20	M	81	12	Disuse syndrome	105	18.7	23	8
Total*	M:18, F: 2	70.7 ±12.5	13.1±2.6	Cerebrovascular: 9, Dysphagia with Aspiration pneumonia: 6, Other: 5	249.5 ±332.8	19.7 ±3.9	26.9 ±2.7	7.2 ±1.1

Psychological measurements

Appetite ratings in after fMRI sessions showed mean values of 7.04 ± 1.12 for iEat food products and 3.89 ± 1.87 for pureed foods. Participants demonstrated significantly greater appetite scores for iEat food products versus pureed foods (p < 0.001, Wilcoxon rank sum test). The median Council on Nutrition Appetite Questionnaire-Japanese (CNAQ-J) score was 28 (IQR: 26.8-29.3), with nine patients classified as at risk of anorexia (score ≤28).

fMRI

**Figure 2:**
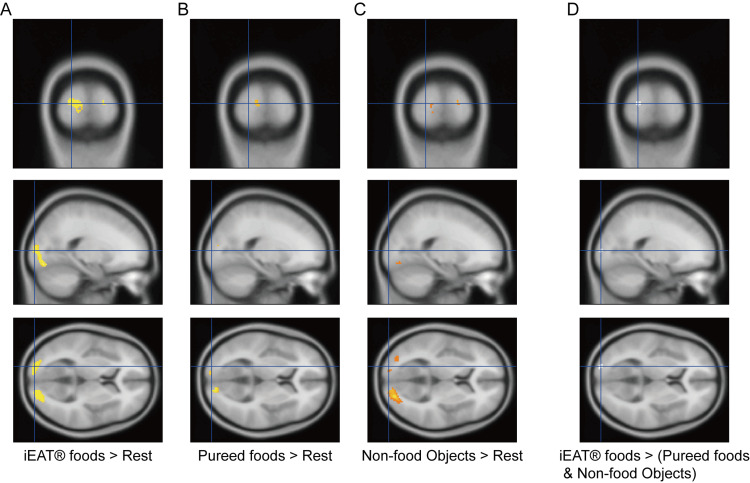
Brain activation patterns during visual stimulus presentation Brain activation patterns during visual stimulus presentation. (A) iEat® food (EN Otsuka Pharmaceutical Co. Ltd, Tokyo, Japan) images, (B) pureed food images, and (C) non-food object images versus resting baseline. (D) Enhanced neural activity in the left middle occipital gyrus for iEat food product stimuli relative to pureed food and non-food object stimuli. Functional activation maps are overlaid onto spatially normalized T1-weighted anatomical scans (FWE-corrected p < 0.05 at voxel and cluster levels). MNI coordinates and statistical parameters are listed in Table 2. NOTE: In the figure, ">" stands for "versus" FWE: Family-Wise Error; MNI: Montreal Neurological Institute

Relative to baseline rest, visual stimuli of iEat food products, pureed foods, and non-food objects produced significantly increased activation in bilateral visual cortex, including the fusiform gyrus (Figures 2A, 2B, and 2C; Table 2). To examine differential brain responses during iEat food item viewing, we analyzed neural activity contrasts between iEat food products versus pureed foods and non-food objects. Whole-brain analysis demonstrated significantly enhanced activation in the left middle occipital gyrus (MNI coordinates: x = -20, y = -96, z = 6; t = 7.81; cluster size k = 17 voxels, FWE-corrected p < 0.05) (Figure 2D; Table 2).

**Table 2:** Coordinates and statistical values of significant brain activity in each contrast Statistical threshold of FWE-corrected p < 0.05 at both voxel and cluster levels. FWE: Family-Wise Error

Laterality	Anatomy	Coordinate (x y z)			t	k
iEat® food products versus the rest						
L	Middle occipital gyrus	-14	-90	-4	11.96	1035
R	Calcarine gyrus	16	-88	-2	11.38	419
R	Inferior occipital gyrus	36	-74	-8	7.80	15
R	Fusiform gyrus	32	-52	-20	7.68	10
Pureed foods versus the rest						
R	Calcarine gyrus	14	-86	-2	12.81	43
L	Inferior temporal gyrus	-44	-62	-10	10.13	47
L	Fusiform gyrus	-34	-48	-22	9.45	47
R	Fusiform gyrus	32	-48	-16	8.19	41
L	Middle occipital gyrus	-24	-84	14	8.18	38
R	Fusiform gyrus	24	-70	-6	7.77	43
Non-food objects versus the rest						
L	Inferior temporal gyrus	-44	-62	-10	17.31	1155
R	Superior occipital gyrus	24	-86	2	14.04	989
L	Calcarine gyrus	-14	-92	-6	11.07	266
L	Middle occipital gyrus	-32	-84	8	9.60	215
R	Fusiform gyrus	32	-54	-20	9.29	181
L	Inferior frontal gyrus	-46	30	12	8.18	14
iEat® food products versus pureed foods and non-food objects						
L	Middle occipital gyrus	-20	-96	6	7.81	17

ROI analysis showed significant activity in the left lingual gyrus (x = -14, y = -90, z = -8; t = 6.30; k = 187 voxels) and right middle occipital gyrus (x = 34, y = -86, z = 8; t = 4.19; k = 7 voxels, FWE-corrected p < 0.05). However, no significant activity was observed in any of the reward system regions examined, including bilateral amygdala, OFC, ventral striatum, and anterior insula.

Additionally, correlation analysis revealed a significant positive relationship between left middle occipital gyrus activity and subjective appetite ratings (r = 0.596, p = 0.006), indicating that greater visual cortex activation was associated with higher appetite scores for iEat food products.

## Discussion

In this study, we used fMRI to examine the effect of visual stimuli from iEat food items on cerebral activation in patients with dysphagia. This fMRI approach provides objective neuroimaging evidence, offering insights into neural mechanisms that behavioral measures alone cannot capture. Whole-brain analysis revealed significantly greater activity in the left middle occipital gyrus during the iEat food product presentation compared to pureed food and non-food object presentation. ROI-based analysis also showed significant activity in the bilateral visual areas. The enhanced visual cortex activity during the iEat food product presentation aligns with the meta-analysis by Tang et al. [22] and the study by Okamoto et al. [13]. Prior research has shown that rewards can increase visual cortex activity [13,22-26]. For instance, Serences demonstrated value-based modulations in the human visual cortex, indicating that the reward value of stimuli can influence visual cortical activity [24]. The increased visual cortex activation in response to iEat food items may reflect their inherent reward value, as their attractive appearance may be perceived as more rewarding than less appealing alternatives. Supporting this interpretation, we found a significant positive correlation between left middle occipital gyrus activity and subjective appetite ratings (r = 0.596, p = 0.006).

Despite significant subjective appetite ratings, patients with dysphagia showed no significant brain reward system activity in any of the examined regions, including bilateral amygdala, ventral striatum, OFC, and anterior insula, contrasting with findings in healthy adults [13,27]. Several factors may contribute to this comprehensive absence of reward system activity. First, the diverse characteristics and variability among the patient group may have influenced the results. In particular, the presence of brain lesions in nine participants with cerebrovascular disease potentially influenced the heterogeneous brain activity patterns. Second, older adults exhibit reduced fMRI signals in the prefrontal cortex and striatum compared to younger adults [28,29], making it more challenging to detect significant activation in these regions. Third, nine patients were at risk for anorexia based on CNAQ-J scores. An fMRI meta-analysis found that patients with anorexia nervosa had lower brain reward system activity compared to healthy subjects [30]. These factors potentially explain the comprehensive lack of brain reward system activity in our dysphagia patient group.

This study has several limitations. First, the patient group's heterogeneity in underlying medical conditions may have influenced neural responses and contributed to the lack of significant reward system activity. Second, the use of a 1.5T MRI scanner potentially limits spatial resolution and signal-to-noise ratio, particularly for small subcortical structures. Third, the gender imbalance in our study population resulted from the available patient demographics during recruitment and our small sample size. Fourth, comparing only iEat food products to pureed food limited the generalizability of our findings. Finally, the lack of healthy age-matched controls limited our ability to elucidate specific neural alterations associated with dysphagia and the potential impact of aging on food-related neural responses. Future research should compare iEat food products with other visually appealing food preparation methods (e.g., molding, piping) and include healthy age-matched controls to comprehensively assess neurological responses to visually enhanced texture-modified foods in patients with dysphagia. Despite these limitations, this study provides valuable insights into neural responses to visually appealing foods in patients with dysphagia.

## Conclusions

This fMRI study demonstrates that patients with dysphagia show significantly enhanced visual cortex activity when viewing visually appealing iEat food products compared to conventional pureed foods, with patients rating iEat food items as significantly more appetizing. Patients did not show significant reward system activation, possibly reflecting the heterogeneous patient population and age-related neural changes. Therefore, future studies comparing responses in healthy age-matched elderly populations would provide more definitive insights into dysphagia-specific neural changes versus age-related alterations. The robust visual cortex response reflects the inherent reward value of visually appealing foods, suggesting that attractive food appearance remains neurologically significant for dysphagia patients. These findings support the clinical use of visually appealing texture-modified foods to potentially improve appetite and nutritional outcomes in dysphagia management.
